# Protein Interactions in *Rhodopseudomonas palustris* TIE-1 Reveal the Molecular Basis for Resilient Photoferrotrophic Iron Oxidation

**DOI:** 10.3390/molecules28124733

**Published:** 2023-06-13

**Authors:** Inês B. Trindade, Maria O. Firmino, Sander J. Noordam, Alexandra S. Alves, Bruno M. Fonseca, Mario Piccioli, Ricardo O. Louro

**Affiliations:** 1 Instituto de Tecnologia Química e Biológica, Universidade Nova de Lisboa, Avenida da República (EAN), 2780-157 Oeiras, Portugal; itrindad@caltech.edu (I.B.T.); snoordam@itqb.unl.pt (S.J.N.);; 2 Magnetic Resonance Center, Department of Chemistry, University of Florence, Via L. Sacconi 6, 50019 Sesto Fiorentino, Italy

**Keywords:** cytochrome *c*, HIPIP, paramagnetic NMR, photoferrotrophism, protein interactions, biological electron transfer, *Rhodopseudomonas*

## Abstract

*Rhodopseudomonas palustris* is an alphaproteobacterium with impressive metabolic versatility, capable of oxidizing ferrous iron to fix carbon dioxide using light energy. Photoferrotrophic iron oxidation is one of the most ancient metabolisms, sustained by the *pio* operon coding for three proteins: PioB and PioA, which form an outer-membrane porin–cytochrome complex that oxidizes iron outside of the cell and transfers the electrons to the periplasmic high potential iron–sulfur protein (HIPIP) PioC, which delivers them to the light-harvesting reaction center (LH-RC). Previous studies have shown that PioA deletion is the most detrimental for iron oxidation, while, the deletion of PioC resulted in only a partial loss. The expression of another periplasmic HiPIP, designated Rpal_4085, is strongly upregulated in photoferrotrophic conditions, making it a strong candidate for a PioC substitute. However, it is unable to reduce the LH-RC. In this work we used NMR spectroscopy to map the interactions between PioC, PioA, and the LH-RC, identifying the key amino acid residues involved. We also observed that PioA directly reduces the LH-RC, and this is the most likely substitute upon PioC deletion. By contrast, Rpal_4085 demontrated significant electronic and structural differences from PioC. These differences likely explain its inability to reduce the LH-RC and highlight its distinct functional role. Overall, this work reveals the functional resilience of the *pio* operon pathway and further highlights the use of paramagnetic NMR for understanding key biological processes.

## 1. Introduction

Photoferrotrophism is proposed to be one of the most ancient types of photosynthesis, which predates oxygenic photosynthesis and is likely to have contributed to the deposition of Precambrian banded iron formations [1,2]. In the ferruginous Archean Ocean, photoferrotrophism enabled microorganisms to grow by using sunlight to drive the coupling of the biogeochemical cycles involving iron and carbon [2,3]. Extant photoferrotrophs are phylogenetically diverse and use both soluble ferrous iron compounds and insoluble iron minerals as electron donors [4]. Whereas soluble ferrous iron can permeate the outer envelope of bacterial cells and can be oxidized by proteins in contact with the energized cytoplasmic membrane, the oxidation of insoluble iron minerals requires the uptake of electrons from outside of the cell in a process called extracellular electron transfer (EET) [5,6,7]. A study investigating the molecular bases for EET identified cytochrome c as key player in this process. In Gram-negative bacteria, protein complexes containing a cytochrome inserted into the cavity of a porin that permeates the outer membrane were identified, whereas in Gram-positive bacteria, these are substituted by molecular wires formed by cytochromes that span the full thickness of the cell wall [8,9,10,11,12].

*Rhodopseudomonas palustris* (*R. palustris*) is one of the most metabolically versatile microorganisms known, and the strain TIE-1 engages in photoferrotrophism [13,14]. This metabolism is sustained by the *pio* operon, which codes for three proteins: PioA and PioB, forming the archetypal cytochrome and porin complex on the outer membrane involved in EET [15], and PioC, which is a high potential iron–sulfur protein (HiPIP). PioC is predicted to be mobile in specialized periplasmic invaginations containing the LH-RC to transfer electrons from PioA to the LH-RC in non-cyclic photosynthesis [14]. In addition to non-cyclic photosynthesis, TIE-1 also engages in cyclic photosynthesis involving the b*c*_1_ complex embedded in the cytoplasmic membrane and mediated by the soluble redox shuttle cytochrome *c*_2_ also present in the periplasm [14].

The photoferrotrophic metabolism is of great interest to study how life and Earth co-evolved through the coupling of biogeochemical cycles sustained by sunlight, and because it can be co-opted for biotechnological processes of low carbon footprint [1,3]. R. palustris has been extensively studied in this regard, and numerous demonstrations of the biotechnological potential of its metabolism have been reported [3,16,17,18,19,20]. In this context, it is important to clarify the final details of the electron-transfer pathway that connects extracellular iron minerals or conducting electrodes with the LH-RC. It was found that the deletion mutation of PioC impairs photoferrotrophic activity but does not abolish it [13,21]. The search for an alternative to PioC led to the identification of the gene *rpal_4085*, which encodes an additional HiPIP protein that is upregulated fourfold in planktonic illuminated cells [14,22]. Although Rpal_4085 was postulated to be able to replace PioC based on its common nature and similar reduction potential, it is unable to donate electrons to the illuminated LH-RC, instead appearing to be involved in the cellular response to divalent metals [14].

To close the knowledge gap regarding the photoferrotrophic metabolism of *R. palustris* TIE-1, in this work, we mapped the interactions and electron transfer processes between PioA, PioC, and the LH-RC. We further showed that PioA can directly transfer electrons to the LH-RC and therefore sustains photoferrotrophism in the absence of PioC. Rpal_4085, despite being a HiPIP with a similar reduction potential at high pH values, presents distinct structural and electronic features that likely explain its inability to substitute PioC.

## 2. Results and Discussion

### 2.1. Probing Photoferrotrophic Iron Oxidation Interactions Using NMR Spectroscopy

To confirm the hypothesis that PioA oxidizes Fe(II) and transfers the electrons to periplasmic protein PioC, which in turn transfers the electrons to the LH-RC, we mapped the interactions between PioA, PioC, and the LH-RC using NMR spectroscopy [13,14].

**Figure 1:**
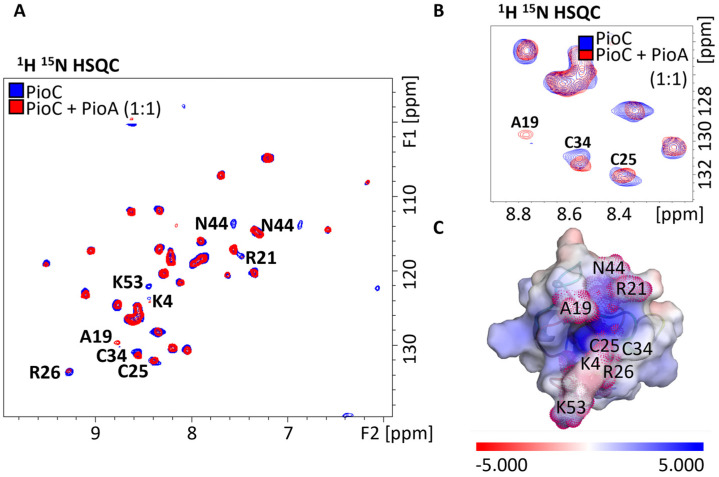
Interactions between PioC and PioA. (**A**) Paramagnetically tailored ^1^H^15^N-HSQC spectra of ^15^N PioC alone (blue) and with PioA (red). (**B**) Both chemical shift changes and line-broadening effects can be observed on HN resonances. (**C**) Protein map of changing resonances in the 3D structure of PioC. Electrostatic surface potential (−5 to +5 kT/e) of PioC (PDB ID: 6XYV) highlighting residues that interact with PioA. Electrostatic surface potentials were calculated using the APBS plugin in PyMOL [27,28].

NMR is a highly sensitive method to probe protein–protein interactions. The binding of an NMR-silent protein (not labeled with ^15^N or ^13^C) to an NMR-visible protein can induce chemical shift changes and/or alter the resonance linewidth of the latter [23,24]. Once the resonances have been assigned, the mapping of chemical shift differences provides residue-specific information on the interface between two proteins. We recently assigned 99.5% of the backbone chemical shifts of PioC NMR spectra using a combination of standard and paramagnetically tailored experiments [25,26]. Upon mixing unlabeled PioA with ^15^N-labeled PioC, spectral changes were observed, confirming the proposed interaction between these two proteins. Using HSQC-AP (heteronuclear single quantum coherence-antiphase) experiment [26], two types of spectral changes were observed: chemical shift changes for residues A19, C25, and C34, and linewidth broadening for residues K4, R21, R26, N44, and K53 (Figure 1A). When we mapped these residues into the three-dimensional structure of PioC together with its electrostatic surface, six of these residues were at the forefront of a positive pocket that constituted the most accessible surface to the 4Fe-4S cluster (Figure 1B,C).

**Figure 2:**
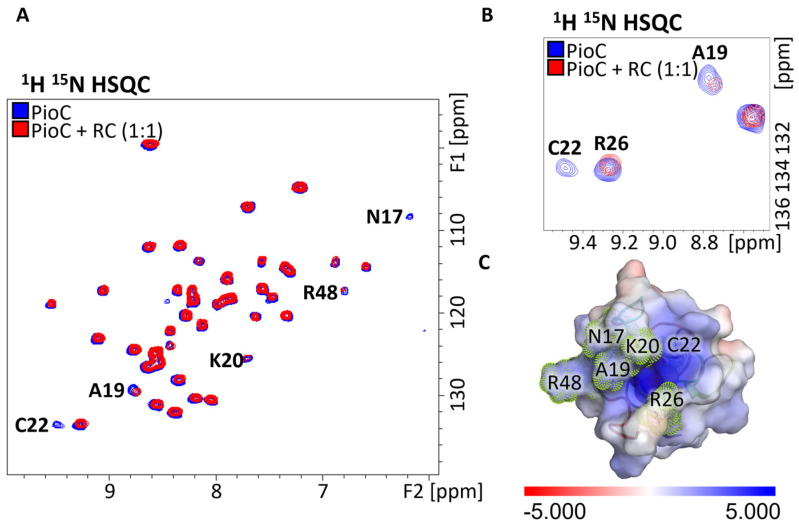
Interactions between PioC and LH-RC. (**A**) Paramagnetically tailored 1H15N-HSQC spectra of 15N PioC alone (blue) and with LH-RC (red). (**B**) Both chemical shift changes and line-broadening effects can be observed on HN resonances. (**C**) Protein map of changing resonances in the 3D structure of PioC. Electrostatic surface potential (−5 to +5 kT/e) of PioC (PDB ID: 6XYV) highlighting residues that interact with LH-RC. Electrostatic surface potentials were calculated using the APBS plugin in PyMOL [27,28].

Using EPR spectroscopy, it was previously observed that PioC is able to transfer electrons to the LH-RC upon sample illumination [14]. To understand which residues were important for the interaction between these proteins, we titrated LH-RC into PioC. Upon mixing LH-RC with PioC, chemical shift changes in residues A19, K20, and R48 and line-broadening effects in residues A19, N17, and C22 (Figure 2B) were observed. Most of the residues involved in this interaction were different (only A19 and C22 were conserved) from the ones that interacted with PioA; however, they still agglomerated at the surface near the access to the 4Fe-4S cluster (Figure 2B,C). 

Overall, the spectral changes observed between PioC and PioA and the LH-RC confirmed the proposed *pio* operon pathway.

### 2.2. Rpal_4085—Why Is It Unable to Substitute PioC?

Having mapped the key interacting residues that allow PioC to mediate electron transfer between PioA and LH-RC, we then aimed to understand why Rpal_4085, despite being an HiPIP, cannot transfer electrons to the LH-RC [14].

**Figure 3:**
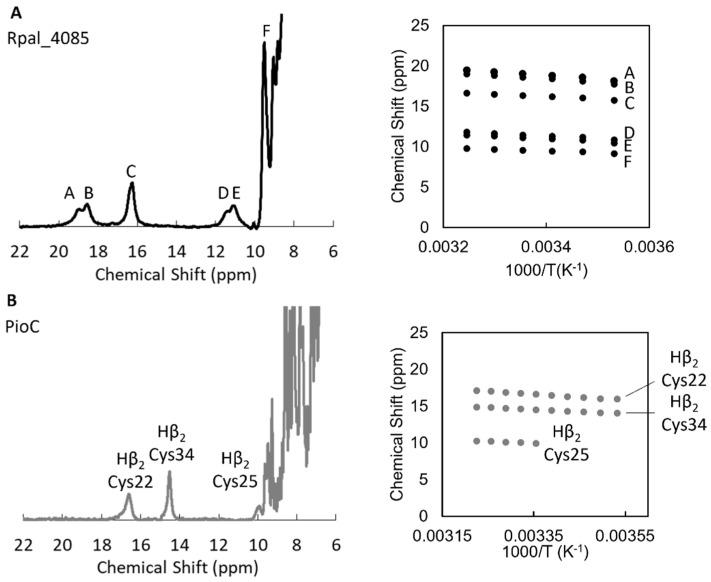
Paramagnetic NMR spectroscopy of PioC and Rpal_4085. (**A**) One-dimensional ^1^H NMR spectra of reduced Rpal_4085 and temperature dependence of hyperfine shift of cysteine β-CH_2_ and α-CH protons (A–F) outside the diamagnetic envelope. (**B**) One-dimensional ^1^H NMR spectra of reduced PioC and temperature dependence of the hyperfine shift of cysteine β-CH_2_ protons outside the diamagnetic envelope.

The 4Fe-4S cluster in the reduced form of HiPIPs is diamagnetic, but at room temperature, excited paramagnetic electronic states are thermally populated due to the small energy gap from the ground state [29,30]. Therefore, the electronic structure of the 4Fe-4S cluster impacts the temperature dependence of hyperfine-shifted signals outside of the protein envelope arising from β-CH2 protons of the cluster-coordinating cysteines. All signals exhibited anti-Curie temperature dependence (i.e., the signals shifted further downfield as the temperature is increased), indicating an increased population of paramagnetic excited states. The similar slope of the temperature dependence of the signals (Figure 3) and the similar range of their values indicated that the electronic structure of the 4Fe-4S cluster in the two proteins was the same, but the difference in chemical shift indicated that the coordination geometry of the cysteines was different [31]. Furthermore, ^1^H-^15^N-HSQC showed that Rpal_4085 appeared in two conformations within the experimental temperature range, leading to a duplication of the signals (Supplementary Figure S1). In our hands, within the different conditions tested (pH from 5.6 to 7.6), the two conformations were always present with similar intensity. This can reflect physiologically relevant states or partial unfolding of the protein [32].

**Figure 4:**
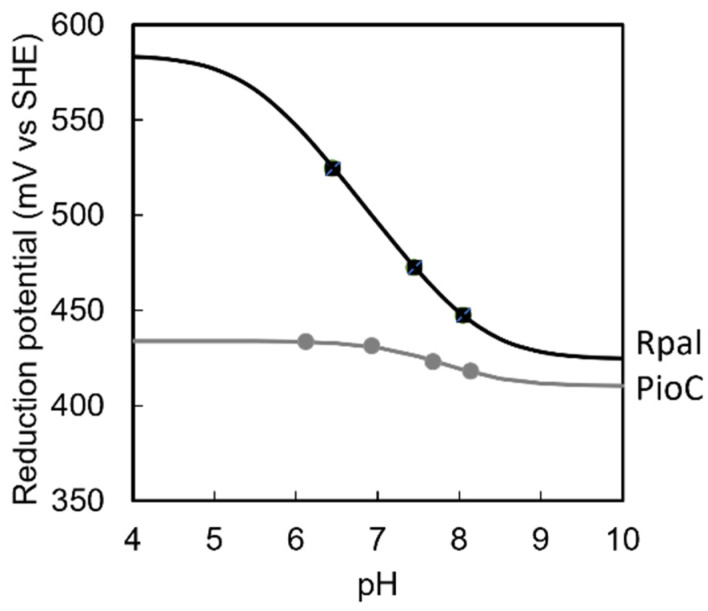
pH dependence of the reduction potentials of PioC and Rpal_4085. The lines were calculated for the coupled transfer of one proton with one electron considering pka_ox_ = 7.6 and pKa_red_ = 8.0 for PioC and pKa_ox_ = 5.5 and pKa_red_ = 8.2 for Rpal_4085 [35]. Of these pK_a_ values, only the pKa_ox_ of PioC was well-defined by the available data, whereas the others represent the upper bounds for the case of the pKa_ox_ of Rpal_4085 and the lower bounds for the pKa_red_ of both proteins that are compatible with the available experimental data. This allows for the calculation of the curves that illustrate the pH dependence of the potentials of the two proteins.

We further investigated whether the differences in cluster geometry were sufficient to reflect differences in the reactivity of these proteins. Thus, the reduction potentials of PioC and Rpal_4085 were measured within the pH range of 6.0 to 8.1 (Figure 4). Rpal_4085 displayed a more positive reduction potential compared to PioC, as previously reported at pH 9 [14]. However, Rpal_4085 had a much more pronounced redox-Bohr effect (Figure 4), with a greater pH dependence of the reduction potential than PioC. This arose from the coupling of electron transfer with the protonation of nearby residues, leading to well-separated pKa_ox_ and pKa_red_ [33]. The consequence is that the reduction potential of these proteins is significantly different within the physiological pH range, with the potential of Rpal_4085 becoming increasingly positive at lower pH values. The potential of Rpal_4085 surpasses that of the reaction center of anoxygenic phototrophs (~430 mV vs. SHE) in the physiological pH range, making electron transfer thermodynamically unfavorable [14]. This may be a design feature arising from the fact that Rpal_4085 is overexpressed in the presence of divalent metal cations, including Mn(II), Fe(II), Co(II), and Ni(II) [14]. Redox-active heavy metal toxicity is mediated by an enhanced Fenton reaction promoted by their reduced states [34]. The higher potential of Rpal_4085 may help keep these metals oxidized, and therefore in a less reactive state. Furthermore, the increased potential at lower pH values enhances the capacity of Rpal_4085 in oxidizing these metals in the conditions in which they tend to be more soluble. 

**Figure 5:**
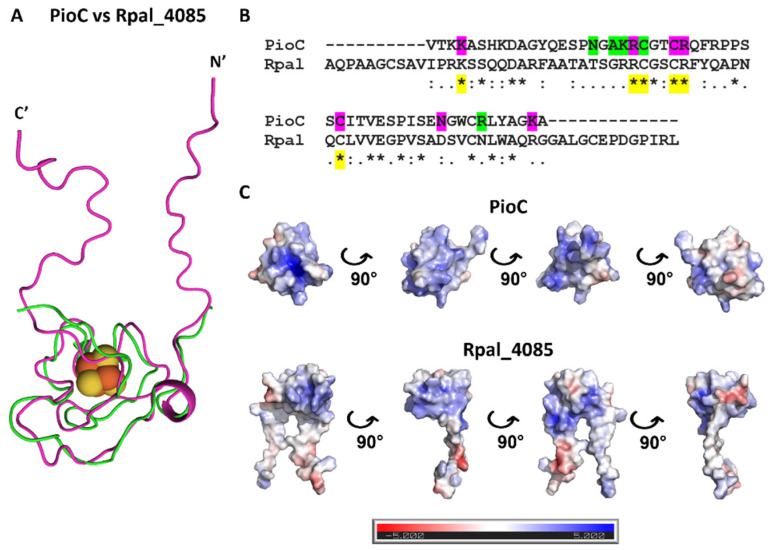
Structural and sequence comparison of PioC and Rpal_4085. (**A**) Structural alignment of PioC (green, PDB ID: 6XYV) and Rpal_4085 (magenta, AlphaFold model). (**B**) Sequence alignment performed with MUSCLE (EMBL-EBI) of PioC and Rpal_4085 where * (asterisk) indicates positions which have a single fully conserved amino-acid residue and : (colon) indicates conservation between groups of strongly similar properties. The PioA-interacting residues are highlighted in magenta, LH-RC interacting residues in green, and conserved residues in yellow. (**C**) Electrostatic surface potential (−5 to +5 kT/e) of PioC (PDB ID: 6XYV) and Rpal_4085 (AlphaFold model). Electrostatic surface potentials were calculated using the APBS plugin in PyMOL [32,37].

We then further investigated the differences between these two HiPIPs by examining the 3D structure and respective electrostatic surface of these proteins. The structure of PioC has been recently determined, and it is accessible through the PDB accession code 6WYV, whereas the Rpal_4085 structure was modeled using the online version of AlphaFold [35,36]. Although the identity of these proteins was 33% and the overall 3D fold was conserved with an RMSD of 0.992 (46 to 46 atoms) for the alignment between residues K4 to A51 of PioC with residues K14 to A61 of Rpal_4085, clear differences were observed (Figure 5). Within the structure of PioC, there was an evident positive pocket that provided access to the 4Fe-4S cluster, whereas in Rpal_4085, this pocket was absent. Furthermore, the predicted model structure of Rpal_4085 showed two regions (N’ and C’ terminus) that extended outwards from the core, resembling two arms, which were completely absent in the structure of PioC (Figure 5A,C). The AlphaFold prediction on these regions was of very low confidence, suggesting either a completely novel fold for these regions or two unfolded regions of Rpal_4085. Furthermore, within the conserved core between PioC and Rpal_4085, the sequence alignment of both proteins showed that at least six of the interacting residues (N17, A19, K20, N44, R48, K53) determined by NMR were absent in Rpal_4085 (Figure 5B). 

### 2.3. PioA: A Direct and Versatile Electron Transfer Pathway to the LH-RC

**Figure 6:**
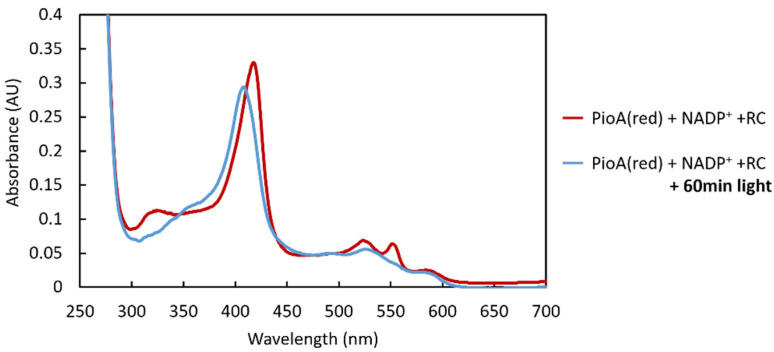
PioA transfers electrons directly to LH-RC. The observed UV-visible spectral changes reflect the oxidation of PioA in the presence of NADP^+^ and the RC upon sample illumination.

Given that Rpal_4085 cannot substitute PioC due to its structural differences and unfavorable thermodynamic properties, we further investigated other alternatives for *R. palustris* to perform photoferrotrophic iron oxidation. It was previously observed that *R. palustris* TIE-1 produces a 34-kDa holo-PioA that exists in two forms: a membrane-associated protein in complex with PioB, and a free soluble periplasmic protein with a reduction potential range extending from +250 to −400 mV vs. SHE [15,38]. These observations, together with the observation that PioA was the most detrimental deletion for Fe(II) oxidation, led to the hypothesis that soluble periplasmic PioA could directly reduce the LH-RC [13]. To test this hypothesis, reduced PioA was incubated in anaerobic conditions with LH-RC and with NADP^+^, which functions as the final electron acceptor. No spectral changes were observed when reduced PioA and NADP^+^ were incubated alone, indicating that PioA did not transfer electrons to NADP^+^. Spectral changes in PioA were only observed upon the addition of catalytic amounts of LH-RC together with sample illumination (Figure 6). These changes were characterized by a shift in the Soret peak from ≈420 nm to ≈409 nm and the disappearance of the α and β peaks at 550 and 520 nm, respectively. These changes indicated that PioA had been oxidized and electron transfer occurred to LH-RC.

## 3. Materials and Methods

### 3.1. Expression and Purification of ^15^N-PioC

PioC was expressed and purified as previously reported [14,25]. Briefly, *E. coli* BL21 DE3 cells were double transformed with pET32h, a plasmid containing the construct thioredoxin-6xHis-thrombin cleavage site-PioC, and with pDB1281, a plasmid that carries the machinery for the assembly of iron–sulfur clusters. The cells were grown at 37 °C in Luria–Bertani (LB) supplemented with 100 µg/mL ampicillin and 35 µg/mL chloramphenicol until an OD_600nm_ of 0.6 before induction with 1.0 mM arabinose and 20 μM FeCl_3_. The cells were again incubated until an OD_600nm_ of 1.0, then harvested and washed in M9 minimal media salts before being resuspended in M9 minimal media with the addition of ammonium sulfate (^15^N, 99%). Once resuspended, the cells were incubated for 1 h before induction with 0.5 mM IPTG. After 4 h, the cells were harvested using centrifugation and stored at −80 °C. The cells were then thawed and resuspended in 50 mM potassium phosphate buffer pH 5.8 with 300 mM NaCl before a three-passage disruption using a French press at 1000 psi. The lysate was ultra-centrifuged at 204,709× *g* for 90 min at 4 °C to remove cell membranes and debris, and the supernatant was dialyzed overnight against a 50 mM potassium phosphate buffer pH 5.8 with 300 mM NaCl before loading in a His-trap affinity column (GE Healthcare, Chicago, IL, USA). The fraction containing Histag-PioC was eluted with 250 mM imidazole and was incubated overnight with Thrombin (GE Healthcare) at 4 °C according to the manufacturer’s protocol for digestion. The final purified PioC (His-tag free) was then concentrated from the flow-through of a second passage through the His-trap column using an Amicon ultra-centrifugal filter (Millipore, Burlington, MA, USA) with a 3 kDa cut-off. The purity of the PioC was confirmed using SDS-PAGE with Blue Safe staining (NZYTech, Lisboa, Portugal), and by UV-visible spectroscopy. For the protein film voltammetry and NMR binding experiments, different aliquot samples of PioC were dialyzed overnight in the appropriate buffers. An extinction coefficient at 390 nm (ε_390nm_ = 16,000 M^−1^cm^−1^) for the 4Fe-4S cluster was used for quantification purposes [39].

### 3.2. Expression and Purification of the LH-RC

*R. palustris* TIE-1 was grown anaerobically on YP medium (10 g/L yeast extract and 20 g/L peptone) at 30 °C under a 36-W white fluorescent lamp for 5 days before the cells were harvested using centrifugation and stored at −80 °C. The cells were thawed and resuspended in 20 mM Tris-HCl pH 8 before a three-passage disruption using a French press at 1000 psi. The lysate was then ultracentrifuged at 204,709× *g* for 90 min, at 4 °C, and the membrane pellets were solubilized using 20 mM Tris-HCl pH 8 with 1% SB-12 (Sulfobetaine-12) and stirred for 1 h at room temperature before the second ultracentrifugation at 204,709× *g* at 4 °C. The supernatant was loaded into a DEAE Sepharose, and the fraction containing LH-RC was eluted with 300 mM NaCl. The purity of the LH-RC was confirmed using SDS-PAGE with Blue Safe staining (NZYTech, Lisboa, Portugal) and UV-visible spectroscopy. An extinction coefficient of 803 nm (ε_800nm_ = 2,570,000 M^−1^cm^−1^) for the bacteriochlorophyll a pigments was used for quantification purposes [40].

### 3.3. Expression and Purification of PioA

PioA was expressed as previously reported [38]. Briefly, *Shewanella oneidensis* MR-1_*Δmtr* cells, which are the deletion mutant of the *mtr* (metal reductase) operon, were electroporated with a pBAD202/D-TOPO expression vector containing a C-terminal Strep II tag together with the *pioA* gene [38]. The cells were grown at 30 °C in LB supplemented with 50 µg/mL of kanamycin until an OD_600nm_ of 0.4–0.6, then induced with 1.0 mM arabinose. After 6 h of growth in the same conditions, the cells were harvested using centrifugation at 10,000× *g* for 10 min at 4 °C. The cells were then resuspended in 50 mM sodium phosphate buffer at pH 8.0 with 300 mM NaCl and a protease inhibitor cocktail (Roche, Basel, Switzerland), DNase I (Sigma-Aldrich, St. Louis, MO, USA), and 1 mM dithiothreitol before a three-passage disruption using a French press at 1000 psi. The lysate was centrifuged at 5500× *g* for 30 min at 4 °C and then ultracentrifuged at 185,000× *g* for 60 min. PioA was purified from the supernatant using a Strep-Tactin XT Superflow Column (IBA-Lifesciences, Göttingen, Germany) followed by a Superdex 75 10/300 GL size exclusion column (GE Healthcare). The eluted fractions were analyzed using SDS-PAGE with heme and Blue-Safe staining (NZYTech) and UV-visible spectroscopy to select the fractions containing pure PioA [41]. The identity of the protein was confirmed using mass spectrometry and N-terminal sequencing. An extinction coefficient of 409 nm (ε_409nm_ = 125,000 M^−1^cm^−1^) per heme was used for quantification purposes [42].

### 3.4. Expression and Purification of Rpal_4085

Rpal_4085 was expressed as previously reported with slight modifications in the purification protocol [14]. Briefly, *E. coli* BL21 DE3 cells were double transformed with pET32h, a plasmid containing the construct thioredoxin-6xHis-thrombin cleavage site-Rpal_4085, and with pDB1281, a plasmid that carries the machinery for the assembly of iron–sulfur clusters. The cells were grown at 37 °C in LB supplemented with 100 µg/mL ampicillin and 35 µg/mL chloramphenicol until an OD_600nm_ of 0.6. Then, the pDB182 plasmid was induced with 1.0 mM arabinose, 20 μM FeCl_3_, and 200 μM cysteine. Once the OD_600nm_ reached 1–1.2, protein expression was induced with 0.5 mM IPTG. After 3 h, the cells were harvested using centrifugation and stored at −80 °C. The cells were then thawed and resuspended in 50 mM potassium phosphate buffer at pH 8.0 with 300 mM NaCl before a three-passage disruption using a French press at 1000 psi. The lysate was ultra-centrifuged at 204,709× *g* for 90 min at 4 °C to remove cell membranes and debris, and the supernatant was dialyzed overnight against a 50 mM potassium phosphate buffer pH 8.0 with 300 mM NaCl before loading in a His-trap affinity column (GE Healthcare). The fraction containing Histag-Rpal_4085 was eluted with 250 mM imidazole and incubated overnight with Thrombin (GE Healthcare) for digestion. After removing the imidazole, a second passage through the His-trap column was performed to remove the His-tagged protein. The final purified Rpal_4085 (His-tag free) was then concentrated from the flowthrough of a second passage through the His-trap column using an Amicon Ultra Centrifugal Filter (Millipore) with a 3 kDa cut-off. The purity of Rpal_4085 was confirmed using SDS-PAGE with Blue Safe staining (NZYTech) and by UV-visible spectroscopy. For the protein film voltammetry and NMR binding experiments, different aliquot samples of Rpal_4085 were dialyzed overnight in the respective appropriate buffers. An extinction coefficient of 390 nm (ε_390nm_ = 16,000 M^−1^ cm^−1^) for the 4Fe-4S cluster was used for quantification purposes [39].

### 3.5. Protein Film Voltammetry of PioC and Rpal_4085

The cyclic voltammetry experiments were conducted at 25 °C inside a Coy anaerobic chamber with a CHI electrochemical analyzer (CHI instruments, Bee Cave, TX, USA) using a three-electrode electrochemical cell configuration with PGE (working electrode), a graphite rod (counter electrode), and an Ag/AgCl 3 M KCl (reference electrode). The PGE working electrode was cleaned with nitric acid and water, and freshly polished before every experiment using 1.0 μm and 0.3 μm of alumina slurry. After washing with bidistilled water, the electrode was left to dry before depositing 10 μL of the protein sample at a concentration of approximately ~250 μM. The sample was allowed to dry on the surface of the electrode before carrying out the experiments at different scan rates in 50 mM potassium phosphate with different pH values. The pH of the buffer was confirmed after each experiment. 

The data were analyzed in Excel following processing using QSoas [25]. All the reduction potentials are reported in mV versus the standard hydrogen electrode (SHE) with the addition of 210 mV to the measured values [43].

### 3.6. ^1^H NMR Temperature Dependence Experiments

A sample of approximately 300 μM of reduced (as purified) PioC and Rpal_4085 in 50 mM potassium phosphate buffer pH 7.6 with 300 mM KCl with 10% D_2_O was used for the ^1^H temperature dependence experiments. The ^1^H NMR experiments were performed using a Bruker Avance II 500 MHz NMR spectrometer equipped with a 5 mm PAQXI probe. A total of 3584 transients were acquired using the super-WEFT (water-eliminated Fourier transform) pulse sequence (180-τ-90-AQ) with 122 ms of recycle time and τ values of 119 ms to dampen the diamagnetic signals and suppress the solvent.

### 3.7. ^15^N PioC: NMR Binding Experiments

The experiments were performed at 298 K using a Bruker AVANCE II+ spectrometer equipped with a 5 mm TCI C/N Prodigy cryoprobe operating at 500 MHz. The reference standard ^1^H-^15^N HSQC experiment was acquired using 72 transients using the pulse sequence hsqcf3gpph1913C from the BRUKER catalog, and the reference paramagnetic ^1^H-^15^N HSQC experiment was acquired as previously described using 256 transients [44]. The duration of each experiment was either 1 h (100 μM PioC) or 3 h (25 μM PioC). Both experiments were performed using ^15^N-labeled PioC in 50 mM potassium phosphate pH 7.0 with 300 mM NaCl (plus 0.01% DDM for binding with LH-RC) with 10% D_2_O. The binding experiments were performed using samples of 100 μM (PioC vs. LH-RC) or 25 μM (PioC vs. PioA) of ^15^N-labeled PioC with increasing amounts of ligand (L): LH-RC or PioA. At each titration point, the spectra were acquired (dark spectra), then the samples were incubated for 30 min ≈10 cm under a 36-W white fluorescent lamp (light spectra). The spectra (standard and paramagnetic) were acquired before and after the illumination step.

### 3.8. Reduction of LH-RC by PioA

The electron transfer experiments of PioA between the LH-RC were carried out inside a Coy anaerobic chamber. The redox state of PioA was followed spectrophotometrically from 250 nm to 700 nm using a Shimadzu 1800 spectrophotometer. PioA was reduced at approximately 0.25 µM in 50 mM sodium phosphate buffer at pH 8.0 with 300 mM NaCl using stepwise additions of sodium dithionite, and excess reducing agent was prevented by monitoring the absorption band of unreacted sodium dithionite at 314 nm [45]. An excess of NADP+ (200×) was added to PioA to serve as an electron sink for the reaction, while catalytic amounts of LH-RC were added to start the reaction. The spectral changes were recorded after every addition and before and after a 60-min illumination step (36 W white fluorescent lamp).

## 4. Conclusions

It was previously proposed by Jiao and co-workers that PioABC proteins are essential for photoferrotrophic iron oxidation in R. palustris TIE-1 [13]. In this work, using paramagnetically-tailored NMR experiments we mapped the interactions of intermediate protein PioC with partners PioA and LH-RC. We also investigated the redox and electronic properties of PioC and Rpal_4085 to mechanistically understand why Rpal_4085, despite being a HiPIP, cannot interact with nor transfer electrons from PioA to LH-RC. Rpal_4085 electrochemical properties are unfavorable for electron transfer to the LH-RC in the physiological pH range because the redox-Bohr effect makes this process thermodynamically unfavorable. Furthermore, in the reduced state, Rpal_4085 exists in two conformations unlike PioC and paramagnetic NMR spectroscopy shows that there are significant differences in the geometry of the cluster ligands. These two conformations may reflect the unknown function of Rpal_4085 and/or a by-product of protein production in conditions that dramatically differ from the physiological ones. By predicting the structure of Rpal_4085 with the use of AlphaFold, we could see a very high degree of conservation of the fold for the core. The most dramatic differences were found in the surface regions that superimpose with those of PioC responsible for the interactions with PioA and the LH-RC [36]. The extended N- and C-terminal regions of Rpal_4085 that are absent in PioC are likely involved in the interaction with its specific physiological partners. Therefore, the evidence collected here elucidates the structural and thermodynamic features that prevent Rpal_4085 from playing a role in photoferrotrophism in R. palustris TIE-1. Evidence which further reinforces the proposal that Rpal_4085 has a different functional role, likely related to the handling of divalent metals in the periplasm [14].

We also found that soluble PioA can transfer electrons directly to the LH-RC. Cytochromes c like PioA are assembled in the periplasmic space of Gram-negative bacteria by specialized maturation systems attached to the inner membrane [15,46]. Therefore, cytochromes that localize to the outer membrane like PioA are present in their final structural configuration in the periplasmic space. A clear precedent for this is reported in the literature for the MtrA decaheme cytochrome from Shewanella oneidensis, which is homologous to PioA and is found both in the membrane and the soluble fraction during purification [37]. The electron transfer between PioA and LH-RC provides a low-activity emergency route for electrons to reach the LH-RC and explains the previously observed retention of photoferrotrophic activity in the absence of PioC [13]. This observation is interesting given that in most purple bacteria the LH-RC includes a multiheme cytochrome which mediates electron transfer between soluble cytochrome c or HiPIP and the special pair of bacteriochlorophylls. In the case of R. palustris TIE-1 this multiheme cytochrome is absent, but the fact that PioA can also transfer electrons directly to the LH-RC suggests conservation of the latter towards the interaction with multiheme cytochromes [47]. Additionally, cytochrome c2 and HiPIP can transfer electrons directly to the LH-RC in cyclic and non-cyclic photosynthesis, respectively. This diversity in the choice of physiological electron donors reveals an additional molecular feature of the metabolic redundancy in this organism already notorious for its remarkable bioenergetic versatility [48].

## Data Availability

Data are available from the corresponding author upon request.

## Supplementary Materials

The following supporting information can be downloaded at: https://www.mdpi.com/article/10.3390/molecules28124733/s1, Figure S1: ^1^H-^15^N HSQC spectra of PioC and Rpal_4085.

## Sample Availability

Materials are available from the corresponding author upon request.

## References

[1] Konhauser K.O., Newman D.K., Kappler A. (2005). The Potential Significance of Microbial Fe(III) Reduction during Deposition of Precambrian Banded Iron Formations. Geobiology.

[2] Camacho A., Walter X.A., Picazo A., Zopfi J. (2017). Photoferrotrophy: Remains of an Ancient Photosynthesis in Modern Environments. Front. Microbiol..

[3] Guzman M.S., Rengasamy K., Binkley M.M., Jones C., Ranaivoarisoa T.O., Singh R., Fike D.A., Meacham J.M., Bose A. (2019). Phototrophic Extracellular Electron Uptake Is Linked to Carbon Dioxide Fixation in the Bacterium *Rhodopseudomonas palustris*. Nat. Commun..

[4] Ilbert M., Bonnefoy V. (2013). Insight into the Evolution of the Iron Oxidation Pathways. Biochim. Biophys. Acta.

[5] Saraiva I.H., Newman D.K., Louro R.O. (2012). Functional Characterization of the FoxE Iron Oxidoreductase from the Photoferrotroph Rhodobacter Ferrooxidans SW2. J. Biol. Chem..

[6] Schink B. (2021). Extracellular Redox Chemistry. Metals, Microbes, and Minerals—The Biogeochemical Side of Life.

[7] Shi L., Dong H., Reguera G., Beyenal H., Lu A., Liu J., Yu H.-Q., Fredrickson J.K. (2016). Extracellular Electron Transfer Mechanisms between Microorganisms and Minerals. Nat. Rev. Microbiol..

[8] Richardson D.J., Butt J.N., Fredrickson J.K., Zachara J.M., Shi L., Edwards M.J., White G., Baiden N., Gates A.J., Marritt S.J. (2012). The “porin-Cytochrome” Model for Microbe-to-Mineral Electron Transfer. Mol. Microbiol..

[9] Edwards M.J., Richardson D.J., Paquete C.M., Clarke T.A. (2020). Role of Multiheme Cytochromes Involved in Extracellular Anaerobic Respiration in Bacteria. Protein Sci..

[10] Costa N.L., Clarke T.A., Philipp L.A., Gescher J., Louro R.O., Paquete C.M. (2018). Electron Transfer Process in Microbial Electrochemical Technologies: The Role of Cell-Surface Exposed Conductive Proteins. Bioresour. Technol..

[11] Faustino M.M., Fonseca B.M., Costa N.L., Lousa D., Louro R.O., Paquete C.M. (2021). Crossing the Wall: Characterization of the Multiheme Cytochromes Involved in the Extracellular Electron Transfer Pathway of Thermincola Ferriacetica. Microorganisms.

[12] Louro R.O., Costa N.L., Fernandes A.P., Silva A.V., Trindade I.B., Fonseca B.M., Paquete C.M. (2018). Exploring the Molecular Mechanisms of Extracellular Electron Transfer for Harnessing Reducing Power in METs: Methodologies and Approaches. Biomass, Biofuels, Biochemicals: Microbial Electrochemical Technology: Sustainable Platform for Fuels, Chemicals and Remediation.

[13] Jiao Y., Newman D.K. (2007). The Pio Operon Is Essential for Phototrophic Fe(II) Oxidation in *Rhodopseudomonas palustris* TIE-1. J. Bacteriol..

[14] Bird L.J., Saraiva I.H., Park S., Calcada E.O., Salgueiro C.A., Nitschke W., Louro R.O., Newman D.K. (2014). Nonredundant Roles for Cytochrome C2 and Two High-Potential Iron-Sulfur Proteins in the Photoferrotroph *Rhodopseudomonas palustris* TIE-1. J. Bacteriol..

[15] Gupta D., Sutherland M.C., Rengasamy K., Meacham J.M., Kranz R.G., Bose A. (2019). Photoferrotrophs Produce a PioAB Electron Conduit for Extracellular Electron Uptake. mBio.

[16] Doud D.F.R., Holmes E.C., Richter H., Molitor B., Jander G., Angenent L.T. (2017). Metabolic Engineering of *Rhodopseudomonas palustris* for the Obligate Reduction of N-Butyrate to n-Butanol. Biotechnol. Biofuels.

[17] Brown B., Wilkins M., Saha R. (2022). *Rhodopseudomonas palustris*: A Biotechnology Chassis. Biotechnol. Adv..

[18] Li M., Ning P., Sun Y., Luo J., Yang J. (2022). Characteristics and Application of *Rhodopseudomonas palustris* as a Microbial Cell Factory. Front. Bioeng. Biotechnol..

[19] Harwood C.S. (2022). *Rhodopseudomonas* *palustris*. Trends Microbiol..

[20] Wang Z., Gao D., Geng H., Xing C. (2021). Enhancing Hydrogen Production by Photobiocatalysis through *Rhodopseudomonas palustris* coupled with Conjugated Polymers. J. Mater. Chem. A.

[21] Jiao Y., Kappler A., Croal L.R., Newman K., Newman D.K. (2005). Isolation and Characterization of a Genetically Tractable Photoautotrophic Isolation and Characterization of a Genetically Tractable Photoautotrophic Fe(II)-Oxidizing Bacterium, *Rhodopseudomonas palustris* Strain TIE-1. Appl. Environ. Microbiol..

[22] Bose A., Gardel E.J., Vidoudez C., Parra E.A., Girguis P.R. (2014). Electron Uptake by Iron-Oxidizing Phototrophic Bacteria. Nat. Commun..

[23] Bax A., Clore G.M. (2019). Protein NMR: Boundless Opportunities. J. Magn. Reson..

[24] Purslow J.A., Khatiwada B., Bayro M.J., Venditti V. (2020). NMR Methods for Structural Characterization of Protein-Protein Complexes. Front. Mol. Biosci..

[25] Trindade I.B., Invernici M., Cantini F., Louro R.O., Piccioli M. (2020). 1H, 13C and 15N Assignment of the Paramagnetic High Potential Iron–Sulfur Protein (HiPIP) PioC from *Rhodopseudomonas palustris* TIE-1. Biomol. NMR Assign.

[26] Trindade I.B., Invernici M., Cantini F., Louro R.O., Piccioli M. (2021). Sequence-Specific Assignments in NMR Spectra of Paramagnetic Systems: A Non-Systematic Approach. Inorg. Chim. Acta.

[27] Konecny R., Baker N.A., McCammon J.A. (2012). IAPBS: A Programming Interface to the Adaptive Poisson-Boltzmann Solver. Comput. Sci. Discov..

[28] DeLano W.L. (2020). The PyMOL Molecular Graphics System.

[29] Bertini I., Briganti F., Luchinat C., Scozzafava A., Sola M. (1991). Proton NMR Spectroscopy and the Electronic Structure of the High Potential Iron-Sulfur Protein from Chromatium Vinosum. J. Am. Chem. Soc..

[30] Poe M., Phillips W.D., Mcdonald C.C., Lovenberg W. (1970). Proton Magnetic Resonance Study of Ferredoxin from Clostridium Pasteurianum. Proc. Natl. Acad. Sci. USA.

[31] Trindade I.B., Coelho A., Cantini F., Piccioli M., Louro R.O. (2022). NMR of Paramagnetic Metalloproteins in Solution: Ubi Venire, Quo Vadis?. J. Inorg. Biochem..

[32] Bertini I., Dikiy A., Luchinat C., Macinai R., Viezzoli M.S., Vincenzini M. (1997). An NMR Study of the 7Fe-8S Ferredoxin from *Rhodopseudomonas palustris* and Reinterpretation of Data on Similar Systems. Biochemistry.

[33] Louro R.O., Catarino T., Salgueiro C.A., LeGall J., Xavier A.V. (1996). Redox-Bohr Effect in the Tetrahaem Cytochrome C3 from Desulfovibrio Vulgaris: A Model for Energy Transduction Mechanisms. J. Biol. Inorg. Chem..

[34] Lloyd D.R., Phillips D.H. (1999). Oxidative DNA Damage Mediated by Copper II, Iron II and ž / Nickel II Fenton Reactions: Evidence for Site-Specific Mechanisms in the Formation of Double-Strand Breaks, 8-Hydroxydeoxyguanosine and Putative Intrastrand Cross-Links. Mutat. Res..

[35] Trindade I.B., Invernici M., Cantini F., Louro R.O., Piccioli M. (2021). PRE-Driven Protein NMR Structures: An Alternative Approach in Highly Paramagnetic Systems. FEBS J..

[36] Jumper J., Evans R., Pritzel A., Green T., Figurnov M., Ronneberger O., Tunyasuvunakool K., Bates R., Žídek A., Potapenko A. (2021). Highly Accurate Protein Structure Prediction with AlphaFold. Nature.

[37] Pitts K.E., Dobbin P.S., Reyes-Ramirez F., Thomson A.J., Richardson D.J., Seward H.E. (2003). Characterization of the Shewanella Oneidensis MR-1 Decaheme Cytochrome MtrA: Expression in Escherichia Coli Confers the Ability to Reduce Soluble FE(III) Chelates. J. Biol. Chem..

[38] Li D.B., Edwards M.J., Blake A.W., Newton-Payne S.E., Piper S.E.H., Jenner L.P., Sokol K.P., Reisner E., Van Wonderen J.H., Clarke T.A. (2020). His/Met Heme Ligation in the PioA Outer Membrane Cytochrome Enabling Light-Driven Extracellular Electron Transfer by *Rhodopseudomonas palustris* TIE-1. Nanotechnology.

[39] Antonkine M.L., Koay M.S., Epel B., Breitenstein C., Gopta O., Gärtner W., Bill E., Lubitz W. (2009). Synthesis and Characterization of de Novo Designed Peptides Modelling the Binding Sites of [4Fe-4S] Clusters in Photosystem I. Biochim. Biophys. Acta Bioenerg..

[40] Saga Y., Hirota K. (2016). Determination of the Molar Extinction Coefficients of the B800 and B850 Absorption Bands in Light-Harvesting Complexes Derived from Three Purple Photosynthetic Bacteria Rhodoblastus Acidophilus, Rhodobacter Sphaeroides, and Phaeospirillum Molischianum by Extraction of Bacteriochlorophyll a. Anal. Sci..

[41] Francis R.T., And J.R., Becker R.R. (1984). Specific Indication of Hemoproteins in Polyacrylamide Gels Using a Double-Staining Process. Anal. Biochem..

[42] Massey V. (1959). The Microestimation of Succinate and the Extinction Coefficient of Cytochrome c. Biochim. Biophys. Acta Bioenerg..

[43] Friis E.P., Andersen J.E.T., Madsen L.L., Bonander N., Moller P., Ulstrup J. (1998). Dynamics of Pseudomonas Aeruginosa Azurin and Its Cys3Ser Mutant at Single-Crystal Gold Surfaces Investigated by Cyclic Voltammetry and Atomic Force Microscopy. Electrochim. Acta.

[44] Ciofi-Baffoni S., Gallo A., Muzzioli R., Piccioli M. (2014). The IR-15N-HSQC-AP Experiment: A New Tool for NMR Spectroscopy of Paramagnetic Molecules. J. Biomol. NMR.

[45] Yousafzai F.K., Eady R.R. (2002). Dithionite Reduction Kinetics of the Dissimilatory Copper-Containing Nitrite Reductase of Alcalegenes Xylosoxidans. The SO2.- Radical Binds to the Substrate Binding Type 2 Copper Site before the Type 2 Copper Is Reduced. J. Biol. Chem..

[46] Verissimo A.F., Daldal F. (2014). Cytochrome c Biogenesis System I: An Intricate Process Catalyzed by a Maturase Supercomplex?. Biochim. Biophys. Acta Bioenerg..

[47] Jackson P.J., Hitchcock A., Swainsbury D.J.K., Qian P., Martin E.C., Farmer D.A., Dickman M.J., Canniffe D.P., Hunter C.N. (2018). Identification of Protein W, the Elusive Sixth Subunit of the *Rhodopseudomonas palustris* Reaction Center-Light Harvesting 1 Core Complex. Biochim. Biophys. Acta Bioenerg..

[48] Nelson K.E., Fraser C.M. (2004). Champions of Versatility. Trends Microbiol..

