# Supplementary material for: Protein Interactions in Rhodopseudomonas palustris TIE-1 Reveal the Molecular Basis for Resilient Photoferrotrophic Iron Oxidation
Source: Molecules. 2023 Jun 13;28(12):4733. doi: 10.3390/molecules28124733 (PMC10304953; doi:10.3390/molecules28124733)
Supplement: Supplementary file 1 [file molecules-28-04733-s001.zip › molecules-2432489-supplementary.docx]

**Supporting Information**

**Protein Interactions in *Rhodopseudomonas palustris* TIE-1 Reveal the Molecular Basis for Resilient Photoferrotrophic Iron Oxidation**

Inês B. Trindade ^1,†^, Maria O. Firmino ^1^, Sander J. Noordam ^1^, Alexandra S. Alves ^1^, Bruno M. Fonseca ^1^,
Mario Piccioli ^2^ and Ricardo O. Louro ^1^

^1^ Instituto de Tecnologia Química e Biológica, Universidade Nova de Lisboa, Avenida da República (EAN),
2780-157 Oeiras, Portugal

^2^ Magnetic Resonance Center, Department of Chemistry, University of Florence, Via L. Sacconi 6, 50019 Sesto Fiorentino, Italy

† Current address: Division of Biology and Biological Engineering, California Institute of Technology,
Pasadena, CA 91125, USA.

| 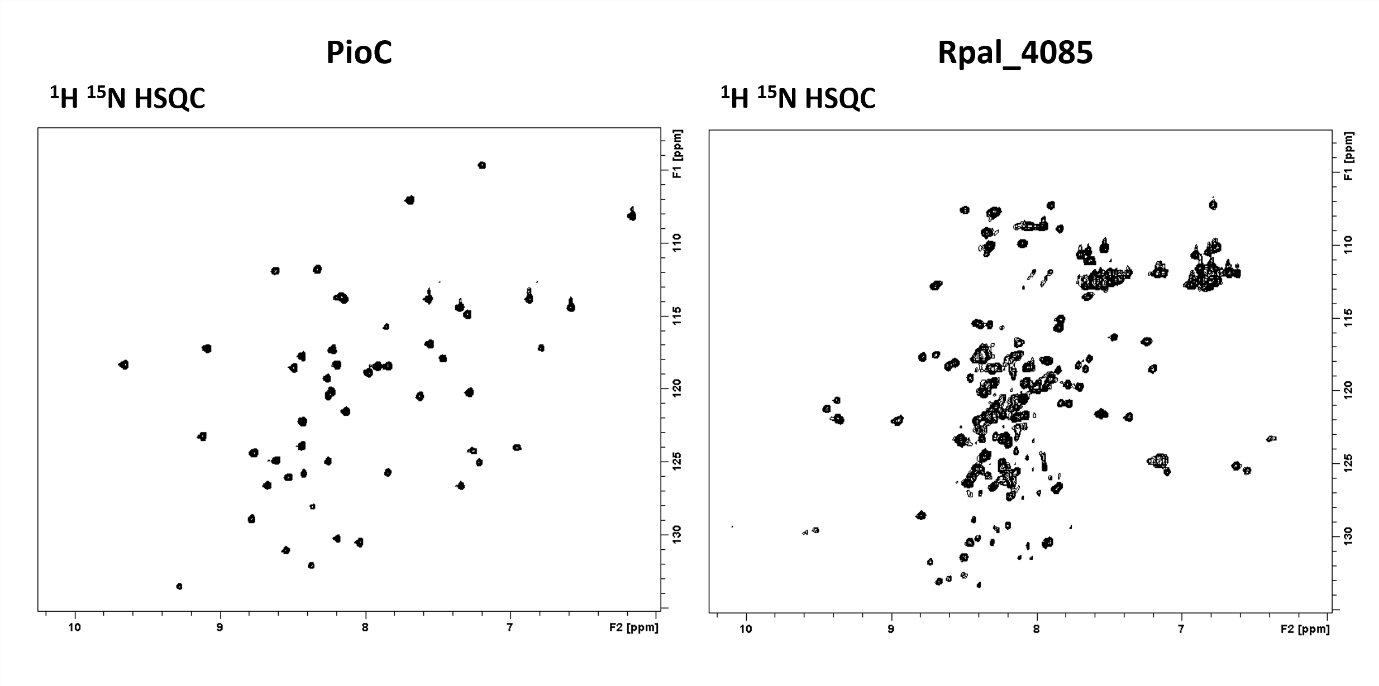 |
| --- |
| **Figure S1.** ^1^H^15^N-HSQC spectra of ^15^N PioC (left) ^15^N Rpal_4085 (right) collected at 900 MHz at 298 K, in 50 mM Potassium Phosphate buffer pH 7.6 with 300 mM KCl with 10 % D_2_O. Both proteins are in the reduced state. Rpal_4085 spectrum shows approximately twice the number of NH peaks as expected from the amino-acid sequence. |
